# Ultra-Short ECG Indices in Patients Presenting with Epigastric Pain—An Orientation Tool for Diagnosis in the Emergency Department

**DOI:** 10.3390/medicina62101845

**Published:** 2026-09-24

**Authors:** Niv Izhaki, Shay Perek, Shiraz Safadi, Ayelet Raz-Pasteur

**Affiliations:** 1 Internal Medicine A Department, Rambam Health Care Campus, Haifa 3109601, Israels_perek@rambam.health.gov.il (S.P.); 2 Emergency Medicine Department, Rambam Health Care Campus, Haifa 3109601, Israel; 3 The Ruth and Bruce Rappaport Faculty of Medicine, Technion-Israel Institute of Technology, Haifa 3200003, Israel

**Keywords:** ultra-short heart rate variability (usHRV), autonomic nervous system (ANS), emergency department (ED), epigastric pain, myocardial infarction (MI)

## Abstract

*Background and Objectives*: Abdominal pain, particularly epigastric pain, is a leading cause of emergency department (ED) visits and encompasses a wide differential diagnosis, ranging from mild conditions such as gastritis and reflux disease to life-threatening etiologies such as acute abdomen and acute myocardial infarction (MI). Epigastric pain is often associated with stress, inflammation, and visceral signaling, processes mediated by the autonomic nervous system (ANS). Changes in ANS activity can be captured by heart rate variability (HRV), a non-invasive measure derived from cardiac rhythm. In this study, we conducted retrospective research to evaluate the prognostic significance of HRV in ED patients presenting with epigastric pain. *Materials and Methods*: Retrospective analysis of patients diagnosed with epigastric pain in the ED at Rambam Health Care Campus in 2014. Ultra-short heart rate variability (usHRV) indices were calculated from a 10 s ED electrocardiogram and correlated with mortality, disease severity, and disease origin. *Results*: 300 patients were included in this study. Due to a very low short-term mortality rate, usHRV could not be reliably assessed for this outcome. The mortality rate throughout the follow-up period of the study was 17.4%, with a median of 8.4 years. No significant association was found between long-term mortality and standard deviation of normal-to-normal intervals (SDNN) or root mean square of successive differences (RMSSD). In multivariate logistic analysis, high RMSSD significantly increased the risk for acute abdomen (RMSSD-adjusted odds ratio (AOR): 1.021, *p*-value: 0.022, Area under the curve (AUC): 73%), while high RMSSD decreased the risk for acute MI (RMSSD AOR: 0.807, *p*-value: 0.026, AUC: 83.6%). *Conclusions*: Ultra-short HRV did not predict long-term mortality in patients presenting with epigastric pain. However, RMSSD was independently associated with both acute abdomen and acute MI. These findings suggest that usHRV parameters may serve as a supportive tool in the ED to aid differentiation between abdominal and cardiac causes of epigastric pain, particularly in high-risk clinical scenarios.

## 1. Introduction

Abdominal pain is a very common complaint in the primary care setting, with a wide differential diagnosis. It is the presenting complaint in 1.5% of office-based visits and in 5% of emergency department (ED) visits. Although most abdominal pain ED visits result in the diagnosis of a benign condition, as many as 10% represent a severe, serious, or life-threatening cause, or require surgery. The location of the abdominal pain together with associated signs and symptoms can help indicate certain causes and narrow the differential diagnosis [1].

Approximately one quarter of ED visits for abdominal pain are attributable to epigastric pain [2]. Epigastric pain can stem from cardiac etiologies such as myocardial infarction (MI) and acute aortic events. Alternatively, it may signal non-cardiac etiologies, including upper gastrointestinal (GI) disorders such as gastroesophageal reflux disease, esophagitis, gastritis, duodenal or gastric ulcer disease, gastric malignancy, and hiatal hernia, as well as pancreatitis and biliary pathology [3,4].

The methods for evaluating abdominal pain, particularly epigastric pain, depend on risk factors, patient history, physical examination, laboratory tests, and imaging studies [1]. Despite the potential for cardiac etiologies, the electrocardiogram (ECG), a simple and accessible tool, is infrequently incorporated into the evaluation of patients presenting with epigastric pain. ECG is a particularly useful tool for measuring heart rate variability (HRV), the fluctuation in the time intervals between adjacent heartbeats. It indexes neuro-cardiac function and reflects heart–brain interactions mediated by the autonomic nervous system (ANS) [5,6,7].

Numerous studies have demonstrated that HRV is a feasible measure of ANS function in relation to cardiac regulation and serves as a reliable predictor of clinical outcomes. Altered HRV has been associated with cardiac-related morbidity and mortality [7] including in patients with chest pain [8] during the acute phase of MI [9,10] and in coronary heart disease [11]. HRV has also been shown to predict mortality in sepsis [12,13,14,15] mesenteric ischemia events [16] and stroke [17].

HRV can be assessed over different time scales: long-term HRV (24 h), short-term HRV (5 min), and ultra-short HRV (usHRV; <5 min) [5,6]. Short-term HRV is considered the gold standard [18]. To assess HRV, several analytical methods can be applied, including time-domain indices, frequency-domain indices and non-linear methods. usHRV research focuses on time-domain indices, as this method requires fewer heartbeats, is stable over short windows and is easier to compute in real time [7,19].

Time-domain indices of HRV quantify the variability in inter-beat intervals (IBIs), defined as the time between successive heartbeats over a specified recording period [5,18]. In a 24 h recording period, the most widely used time-domain analytical measures are the standard deviation of normal-to-normal (NN) IBIs (SDNN), which reflects overall variability, and the root mean square of successive differences between normal heartbeats (RMSSD), which captures short-term beat-to-beat variability [19]. Assessment of usHRV time-domain indices revealed that even a single 10 s RMSSD measurement provides valid and accurate HRV parameters [20]. RMSSD from usHRV has also been found to provide prognostic input in the ED, in various clinical settings [21,22].

In the ED, there is a clear clinical need for a simple, non-invasive tool to evaluate patients presenting with epigastric pain, as this symptom may reflect underlying cardiac pathology. Despite the established prognostic value of HRV across a range of acute and critical conditions [23], no studies to date have examined HRV specifically in relation to epigastric pain. Therefore, in this study, we aim to describe usHRV in ED patients presenting with epigastric pain and to evaluate its prognostic significance.

## 2. Methods

### 2.1. Study Design

Patients admitted to Rambam Health Care Campus ED throughout 2014 and diagnosed with epigastric pain were retrospectively enrolled in the study. Admission ECG recordings were manually checked, and those that contained premature ventricular beats, premature supraventricular beats, atrial fibrillation, or conduction blocks that could potentially affect accurate measurement of HRV were excluded from the study. The study was approved by the Helsinki committee at Rambam Health Care Campus. Since all patient data were collected retrospectively, informed consent was not required. Due to potential effects on HRV indices, patients known to be receiving either beta-blockers or calcium channel blockers were excluded from the study. Patients or the public were not involved in the design, conduct, reporting, or dissemination plans of our research.

### 2.2. Data Collection

Anonymized patient data regarding age, sex, ethnicity, and previous medical diagnoses were collected using the MDClone software (Version 6). Clinical and laboratory admission parameters were obtained, including mean arterial pressure (MAP), heart rate (HR), oxygen saturation (SpO_2_), complete blood count, biochemistry, blood gases, and troponin and brain natriuretic peptide (BNP) levels. Data regarding imaging, surgical procedures, cardiac intervention, and endoscopy were collected manually through review of electronic medical records using the PROMATHEUS system. These included brain, chest and abdominal computed tomography (CT), abdominal ultrasound (US), percutaneous coronary intervention (PCI), pathology reports and procedure files.

### 2.3. ECG and HRV Analysis

All patients arriving at the Rambam Health Care Center ED and diagnosed with epigastric pain underwent an immediate electrocardiogram utilizing the LAN Green model (Norav Medical, Yokne’am, Israel). The procedure was performed within the non-ambulatory wing of the ED, while patients lay motionless in the supine position for at least 30 s. Prior to the examination, patients remained still during preparation for more than 1 min. ECG electrodes were placed in standard anatomical positions according to routine clinical procedures. The actual ECG capture required patients to remain completely still for an additional 10 s. In cases where the ECG quality was subpar, a repeat study was performed immediately. Ultimately, patients whose ECG studies were partially incomplete or of low quality were excluded from our analysis. A dedicated ECG viewing program (Resting ECG version 5.62, Norav Medical) was utilized to visualize the resting ECG files. This software automatically measured the PR and QRS interval durations and calculated the QT interval duration using the Bazett equation. Subsequently, the ECG files were analyzed with a customized version of heart rate variability (HRV) analysis software capable of importing and evaluating 10 s recordings (HRV version 5.62, Norav Medical). These programs enabled the automatic computation of ultra-short-term HRV (usHRV) parameters. Additionally, all ECGs were manually inspected, and recordings exhibiting disturbances—such as excessive noise, sudden baseline instability, or low resolution—were excluded. ECGs demonstrating excessive premature ventricular or supraventricular activity (e.g., atrial fibrillation, atrial flutter, atrial tachycardia), as well as advanced atrioventricular conduction abnormalities (e.g., second-degree atrioventricular blocks, complete heart block, or other high-degree conduction defects), were also excluded. The analysis focused on time-domain HRV indices, specifically SDNN and RMSSD, as continuous variables.

### 2.4. Study Endpoints

The primary endpoint of the study was to evaluate the prognostic significance of HRV parameters in predicting short-term (i.e., 30 and 90 days) and long-term (i.e., survival analysis) mortality among patients presenting with epigastric pain. Survival analysis was based on the ministry of health national death index, which documents the date of death (i.e., all-cause mortality). Patients without a date of death during data extraction were considered alive and therefore were censored. The follow-up interval was calculated based on the ED arrival date and either the date of death or the censoring date. The secondary endpoint of the study was to evaluate the association between HRV parameters and disease severity, defined as a new diagnosis of biopsy-proven cancer, acute abdomen (defined as a medical condition requiring emergent surgical intervention, including: bowel obstruction, mesenteric event, incarcerated hernia, acute appendicitis, bowel perforation), upper GI bleeding, or acute MI (defined as either non-ST elevation or ST elevation MI)—all diagnosed during the index admission. Patient data related to primary and secondary endpoints of the study were collected using the MDClone software or manually obtained from the medical records of participants.

### 2.5. Statistical Analysis

All statistical analyses were performed using R (v4.0.3; R Core Team, 2020). Continuous variables are presented as mean ± standard deviation (SD), or median with interquartile range (IQR), depending on variable distribution. Categorical variables are presented as absolute numbers and percentages. Group comparisons were performed using Student’s *t*-test or Mann–Whitney U test for continuous variables and Fisher’s exact test for categorical variables. Survival analysis was conducted using Cox proportional hazards regression to characterize the relationship between risk factors and outcomes. Results are presented as adjusted odds ratios (AORs) with a confidence interval (CI) of 95%. Survival curves were generated using Kaplan–Meier plots, and regression model performance was assessed with receiver operating characteristic (ROC) curves and the area under the curve (AUC) with 95% CI based on bootstrapping internal validation. Variables showing statistical significance or a trend (*p* < 0.10) in univariate analyses were included in multivariate logistic regression. Due to its importance in HRV assessment, heart rate was introduced as a mandatory covariate. All tests were two-sided, with *p* < 0.05 considered statistically significant.

In addition, in order to contend with the limited number of outcome events, penalized regression (e.g., Firth logistic regression) as well as bootstrapping internal validation (with 1000 replicates) were carried out. Further, an optimism-corrected AUC was calculated.

## 3. Results

### 3.1. Study Design and Patient Selection

A total of 720 patients were admitted to Rambam Health Care Campus in 2014. A 10 s ECG recording on admission was not available for 89 patients. ECG recordings were incompatible with the HRV analysis software for 67 patients. Arrhythmia was detected on the admission ECG recordings of 193 patients. 71 patients were receiving beta-blockers or calcium channel blockers. Consequently, 420 patient cases were excluded from the study. Ultimately, 300 patients were included in the study (Figure 1).

### 3.2. Baseline Characteristics and Patient Work-Up

The baseline characteristics were collected for all study patients and stratified by long-term survival status, as presented in Table 1.

Diagnostic and therapeutic procedures were performed in a subset of patients. Gastroscopy and colonoscopy were undertaken in 19 and 4 patients, respectively. PCI with stent placement was performed in 6 patients. A total of 88 imaging studies were performed, including 10 head CT scans, 11 chest CT scans, 29 abdominal CT scans and 38 abdominal US scans. Overall, 60 patients were classified as having severe disease, including 15 with newly diagnosed cancer, 13 with acute abdomen (including 3 peritonitis, 2 incarcerated hernias, 2 severe colitis, 2 bowel obstruction, 1 paralytic ileus, 1 cholecystitis with perforation, 1 perforated appendix, 1 mesenteric event), 29 with upper GI bleeding, and 6 with acute MI (including 4-STEMI, 2-Non STEMI). Notably, 3 newly diagnosed cancer patients also suffered from acute abdomen (2 with bowel obstruction and 1 with mesenteric event).

### 3.3. HRV as a Predictor of Short- and Long-Term Mortality in Epigastric Pain Patients

Due to the low number of deaths in 30 days (1 patient) and 90 days (2 patients), short-term mortality could not be reliably analyzed. Therefore, survival analysis focused on long-term mortality. COX regression was performed to examine the correlation between HRV and long-term mortality.

Over a median follow-up of 8.4 years (IQR 8.1–8.6), 53 patients (17.6%) died. Univariate survival analysis was performed to compare patients who survived 8 years after an epigastric pain diagnosis with those who did not. No HRV measure was significantly associated with 8-year survival. Specifically, SDNN (HR: 1.010 (95% CI 0.997–1.023), *p*-value = 0.120) and RMSSD (HR: 1.009 (95% CI 0.998–1.020), *p*-value = 0.095) did not differ significantly between survivors and non-survivors. Multivariate analysis was not performed due to the lack of statistical significance in the univariate results.

Univariate logistic regression analysis was performed to examine whether usHRV variables were predictive of new oncological diagnoses or upper GI bleeding. Neither SDNN nor RMSSD showed statistically significant associations with either outcome.

### 3.4. HRV as a Predictor of the Development of Acute Abdomen

13 out of 300 patients (4.3%) were diagnosed with acute abdomen. RMSSD was found to be statistically significant in both univariate and multivariate analyses for predicting acute abdomen. However, SDNN was not found to have statistically significant correlations with acute abdomen. Adjusting for age and heart rate, each 1 ms increase in RMSSD was associated with a 2.3% higher probability of acute abdomen (Figure 2 and Table 2). Model summary: *p*-value 0.005, McFadden R^2^ 0.118, Nagelkerke R^2^ 0.138.

Due to the limited number of outcome events (i.e., events per variable 4.33), the following additional analyses were carried out. Assessment of Firth logistic regression revealed relatively similar results (Age—AOR 0.947 (0.906–0.984), *p*-value 0.005; Pulse—AOR 0.993 (0.952–1.028), *p*-value 0.721; RMSSD—AOR 1.021 (1.004–1.041), *p*-value 0.016).

Internal validation with 1000 bootstrapped replicates demonstrated mixed results (Age—AOR 0.943 (0.891–0.982); Pulse—AOR 0.991 (0.954–1.023); RMSSD—AOR 1.023 (0.988–1.040)), while the optimism-corrected AUC was 0.706.

### 3.5. HRV as a Predictor of the Development of Acute MI

6 out of 300 patients (2%) were diagnosed with acute MI. RMSSD was found to be statistically significant in both univariate and multivariate analyses for predicting acute MI. Adjusting for heart rate and glucose, each 1 ms increase in RMSSD was associated with a 24% lower probability of acute MI. SDNN showed an inverse correlation with acute MI, although this did not reach statistical significance. The combined area under the curve (AUC) for both variables was 0.940, while the AUC for RMSSD alone was 0.836 (Figure 3 and Figure 4 and Table 3). Model summary: *p*-value < 0.001, McFadden R^2^ 0.416, Nagelkerke R^2^ 0.440.

Due to the limited number of outcome events (i.e., events per variable 2), the following additional analyses were carried out. Assessment of Firth logistic regression revealed relatively similar results (Pulse—AOR 1.046 (1.005–1.086), *p*-value 0.034; Glucose—AOR 0.897 (0.814–0.964), *p*-value 0.001; RMSSD—AOR 0.808 (0.620–0.948), *p*-value 0.007).

Internal validation with 1000 bootstrapped replicates demonstrated comparable results (Pulse—AOR 1.061 (1.000–1.193); Glucose—AOR 0.878 (0.704–0.950); RMSSD—AOR 0.739 (0.582–0.965)), while the optimism-corrected AUC was 0.942.

Notably, troponin level (ng/mL) was not found to be correlated with acute MI—OR 0.001 (95% CI 0–1.06); *p*-value 0.87).

## 4. Discussion

Epigastric abdominal pain is a common diagnosis in the ED that can reflect serious abdominal or cardiac pathology. In this study, we assessed time-domain usHRV indices as exploratory, hypothesis-driven prognostic and diagnostic predictors in this patient population. We did not find a correlation between HRV and long-term mortality, which was the primary endpoint. We observed that usHRV time-domain measures were significantly associated with underlying causes of epigastric pain, specifically acute abdomen and acute MI. Higher RMSSD values were associated with acute abdomen, whereas lower RMSSD values were associated with acute MI. These findings suggest that RMSSD may serve as a supportive tool in the ED to help differentiate abdominal from cardiac causes of epigastric pain.

Previous studies have assessed the association between HRV analyses and specific acute abdominal pathologies. For instance, a reduction in HRV was observed prior to the onset of overt clinical sepsis in a porcine model of peritonitis-induced progressive septic shock [23]. Several HRV parameters are reduced in non-surviving septic patients, and low SDNN has been identified as a risk factor for death in septic patients, even after adjusting for severity scores [13,15]. In addition, a recent study examining non-acute causes for epigastric pain reported that gastroesophageal reflux disease (GERD) and functional gastroesophageal diseases (FGIDs) are associated with impaired autonomic function, characterized by suppressed vagal tone and overactive sympathetic tone, which can be assessed noninvasively using HRV [24]. However, these studies did not use HRV analyses to determine the source of epigastric pain.

Previous studies have shown that low SDNN is associated with an increased mortality risk following acute MI [8,25,26,27,28,29]. One study also found that reduced HRV is associated with in-hospital complications in patients with acute MI [8]. However, these studies evaluated HRV after acute MI diagnosis and did not examine its role as a predictive tool. Moreover, these studies did not examine atypical presentations of acute MI, such as epigastric pain, which are more common in female and elderly patients, and their correlation with HRV analyses. Accordingly, the current study addresses this gap by utilizing time-domain HRV measures, in combination with glucose levels, to identify epigastric pain as an atypical presentation of cardiac source.

The vagus nerve is a major component of the parasympathetic nervous system and plays a critical role in controlling cardiac, pulmonary, and GI function. Its influence in the context of acute abdomen relates primarily to its effects on heart rate, digestion, and reflexes that arise in response to pain or visceral irritation. Epigastric pain is often closely associated with vagal nerve activity due to the vagus nerve’s key role in controlling digestive functions and its involvement in pain modulation from the GI. Conditions that stimulate or irritate the stomach or esophagus may activate the vagus nerve, leading to a complex interplay of digestive and autonomic symptoms that can be reflected in HRV measures.

Vagal activation may also accompany cardiovascular pathology. Myocardial ischemia can present with epigastric pain due to shared autonomic pathways and referred pain mechanisms, particularly in women and elderly patients.

An unexpected finding of our study demonstrates that elevated glucose levels are associated with a decreased risk of myocardial infarction. This result stands in direct contradiction to the extensive body of scientific literature, which firmly establishes hyperglycemia as a major cardiovascular risk factor and a predictor of a poorer prognosis and reduced cardiac function following an acute event [30]. It is possible that the observed association is not causal, but rather stems from unmeasured confounding variables. For instance, patients with chronically elevated glucose levels (such as diagnosed diabetics) often receive more aggressive prophylactic medical management. The use of cardioprotective therapies—including statins, antiplatelet medications, or novel antidiabetic medications (such as SGLT2 inhibitors or GLP-1 receptor agonists)—has a proven protective effect on the cardiovascular system. This pharmacological intervention may mask the detrimental effects of glucose, leading to an observed reduction in risk within the study cohort.

This study introduces a novel methodological approach by utilizing usHRV time-domain analyses as a non-invasive indicator of vagal activity to assess the source of epigastric pain, particularly in cases involving critical diagnoses with significant mortality risk, such as acute abdomen and acute MI.

Despite these findings, several limitations should be acknowledged. Firstly, this was a single-center retrospective analysis. Secondly, for the assessment of secondary outcomes, 228 patients discharged from the ED were lost to follow-up due to restrictions on accessing their ambulatory files as per Helsinki guidelines. Although the patients were lost to follow-up, available data indicate that none died or returned to the emergency department of our center. Thirdly, the subgroup analysis of patients diagnosed with acute MI included only six patients, which limits the strength of the analysis. Exclusion of patients receiving beta-blockers or calcium channel blockers may have led to this modest subgroup sample, as patients with coronary heart disease regularly require these chronic medications. Thus, a spectrum bias may have been introduced. The exclusion of more than half of patients originally considered for this study may have also led to a selection bias. Finally, the term “epigastric pain” may be interpreted differently by individual physicians, potentially introducing variability in patient classification.

## 5. Conclusions

In conclusion, vagal nerve function, quantified with usHRV parameters and specifically RMSSD, might have some potential when incorporated in a clinical decision (diagnosis) ED tool. This study focused on its exploratory role, which should be complementary to well-established clinical, laboratory and electrocardiographic markers. Future prospective studies should determine whether RMSSD provides additional diagnostic information beyond these established parameters.

## Figures and Tables

**Figure 1:**
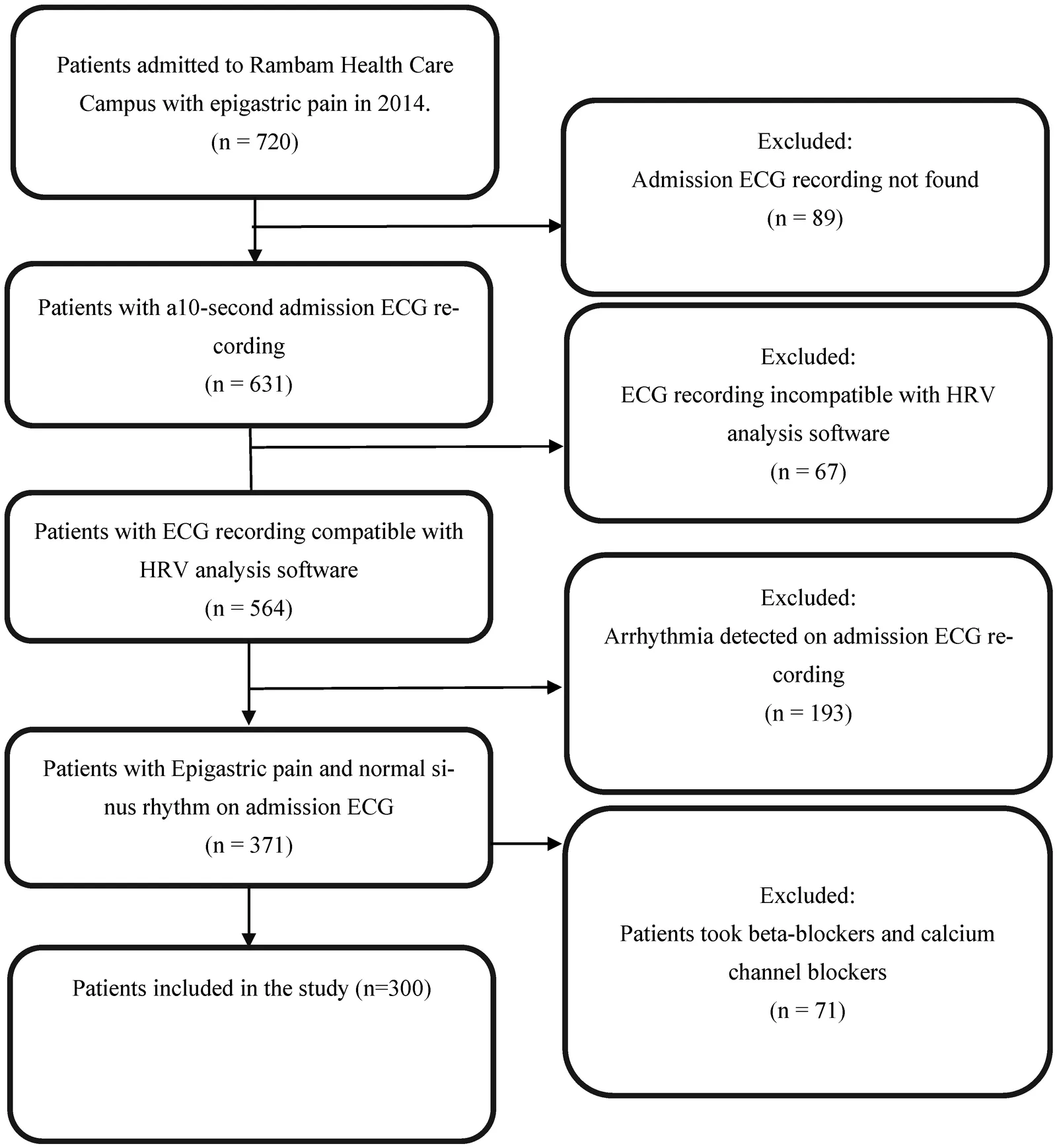
Flowchart of patient selection.

**Figure 2:**
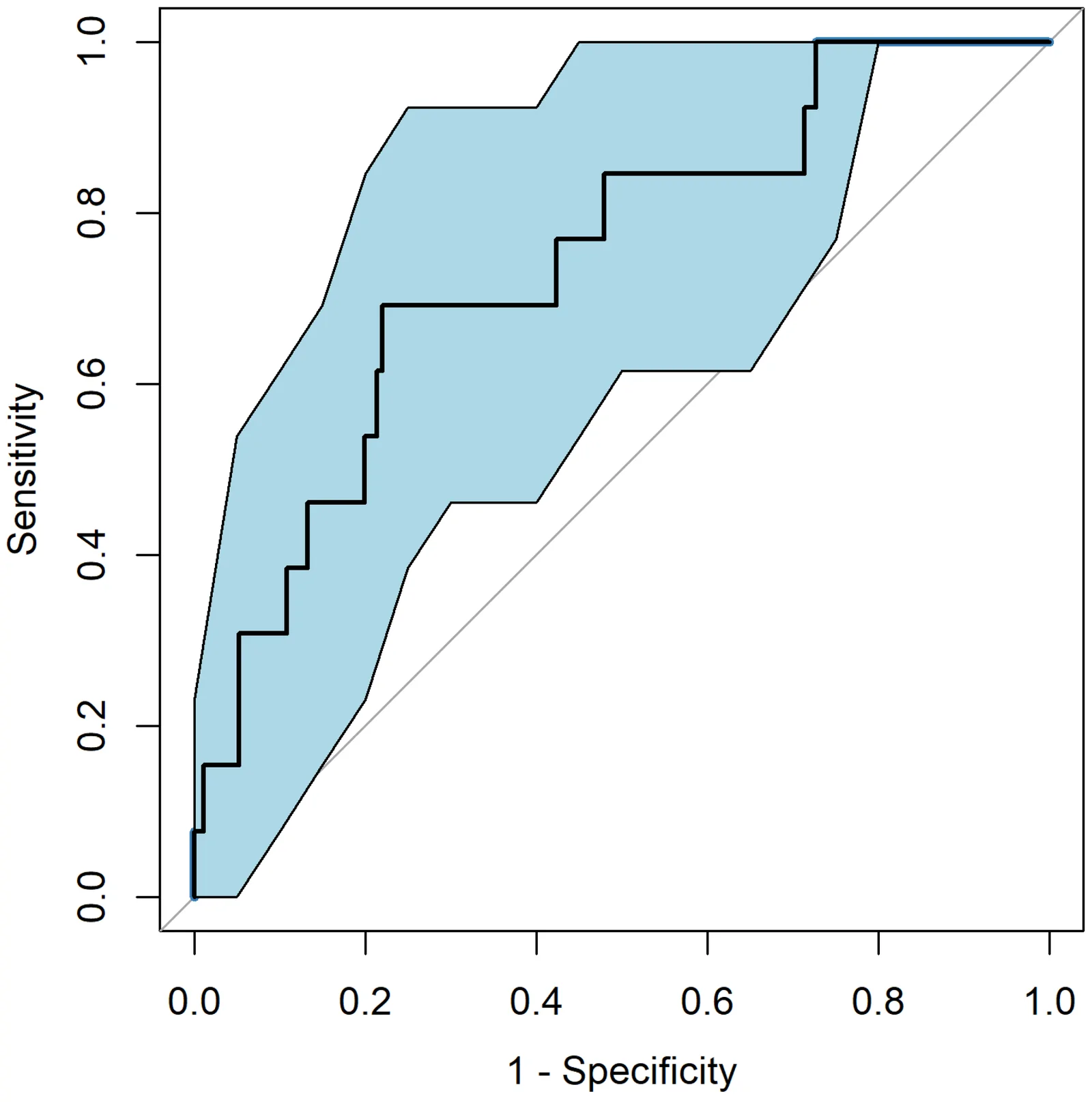
Receiver operating characteristic (ROC) curve of the multivariable logistic regression model for prediction of acute abdomen. AUC was 0.744 (95% CI 0.604–0.883).

**Figure 3:**
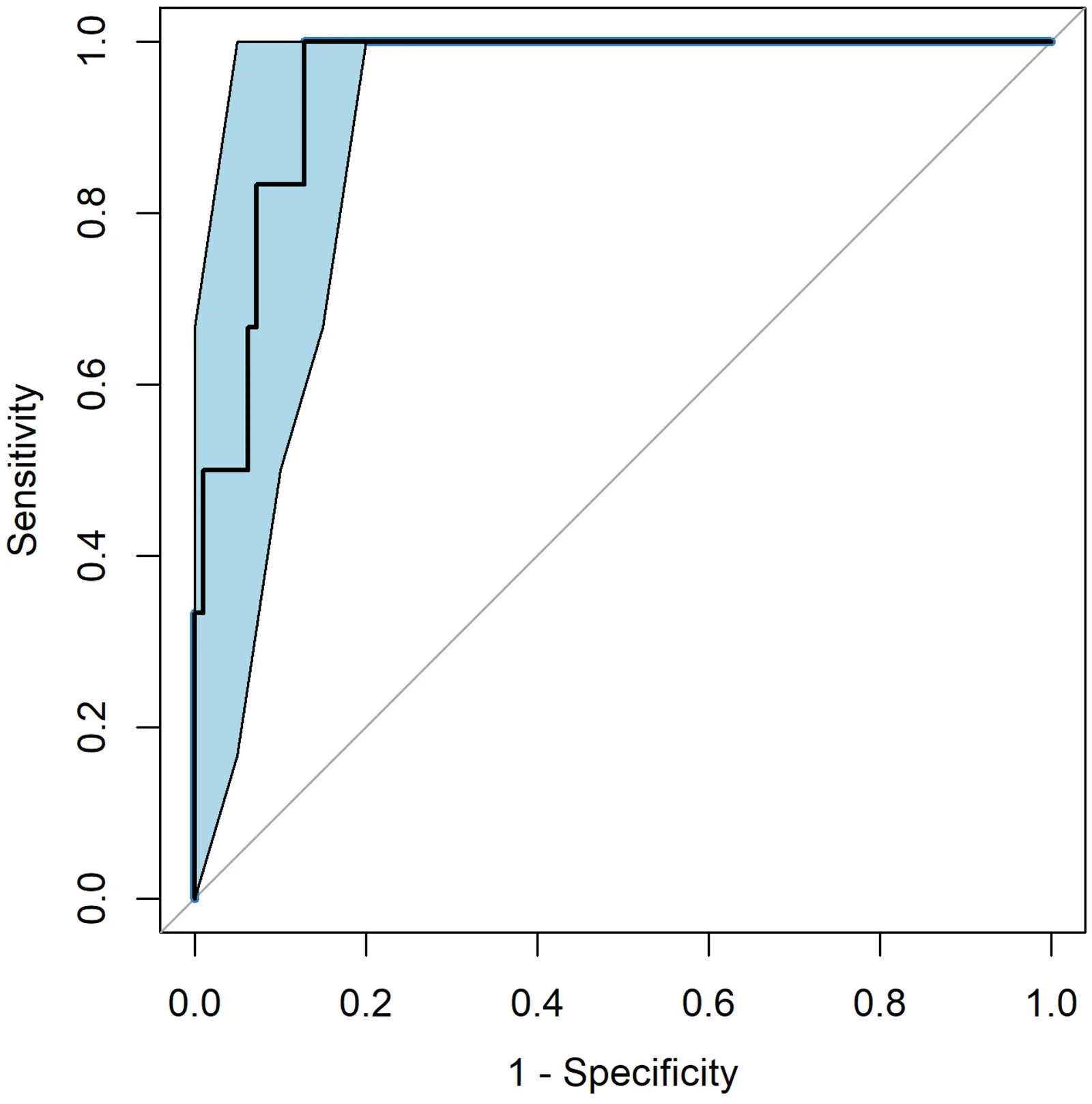
Receiver operating characteristic (ROC) curves for prediction of acute myocardial infarction (MI). The combined multivariable model demonstrated an AUC of 0.955 (95% CI 0.911–0.998).

**Figure 4:**
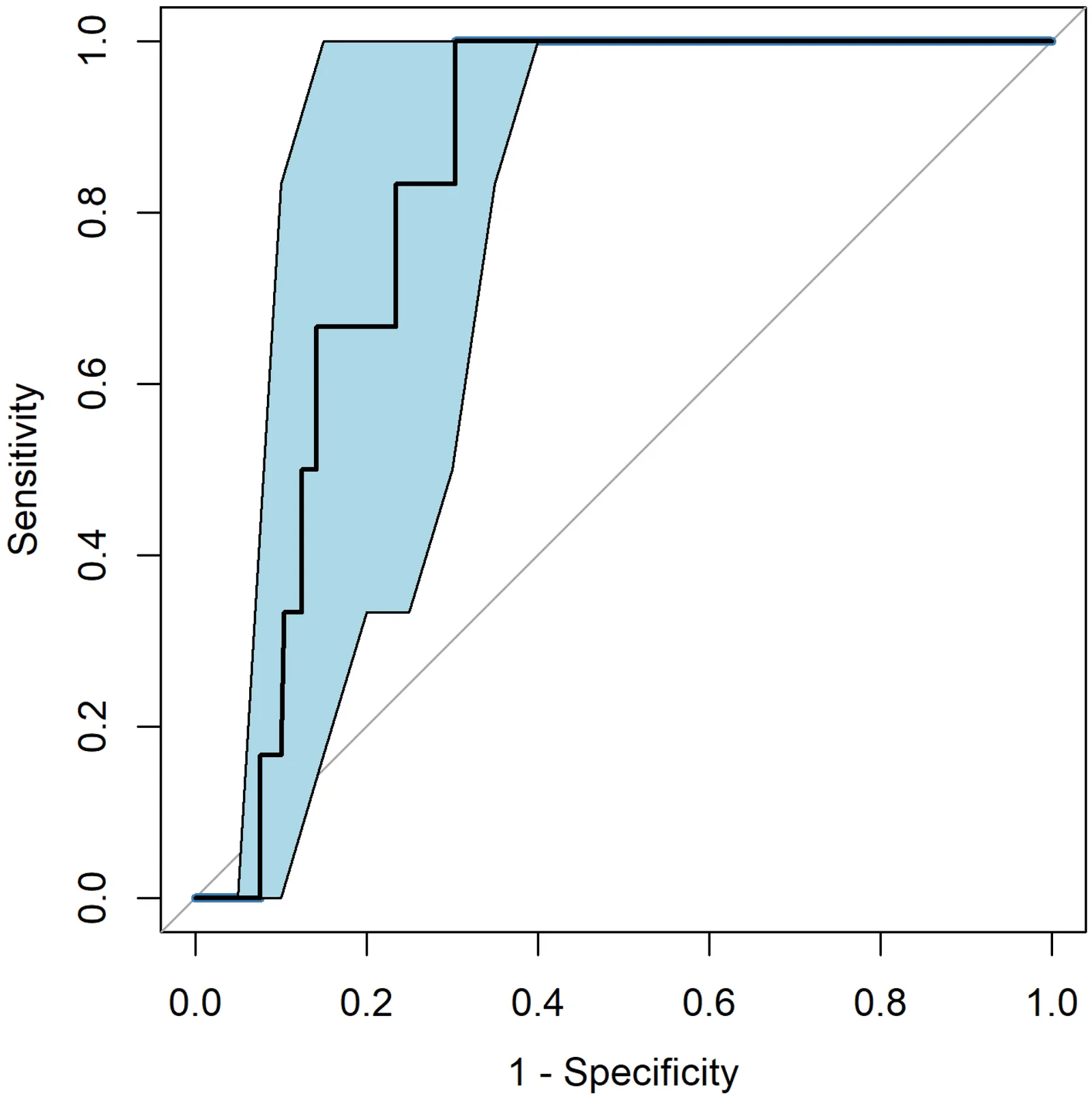
Receiver operating characteristic (ROC) curves for prediction of acute myocardial infarction (MI). RMSSD alone yielded an AUC of 0.836 (95% CI 0.758–0.915).

**Table 1:** Baseline characteristics (*n* = 300) stratified by long-term survival status.

	Survival Status—Censored (*n* = 247)	Survival Status—Deceased (*n* = 53)	*p*-Value
Age (years)	51 (41–64)	76 (65–80)	<0.001 *
Gender (male; %)	108 (44%)	19 (36%)	0.341
BMI (kg/m^2^)	27.2 (24.6–30.9)	27.7 (24.2–31.3)	0.982
Hospitalized (%)	51 (21%)	22 (42%)	<0.001 *
**Vital signs upon ED arrival**			
Systolic blood pressure (mmHg)	137 (123–157)	159 (137–182)	<0.001 *
Diastolic blood pressure (mmHg)	77 (70–87)	80 (74–85)	0.249
Pulse (BPM)	80 (70–87)	81 (71–89)	0.545
Saturation (%)	98 (96–99)	97 (95–98)	0.005 *
Temperature (°C)	36.8 (36.6–36.9)	36.7 (36.5–36.9)	0.436
**Labs upon ED arrival**			
WBC (10^9^/L)	8.9 (7.3–10.7)	8.5 (6.8–11.6)	0.853
Hemoglobin (g/dL)	13.5 (±1.5)	11.9 (±2.0)	<0.001 *
Platelet (10^9^/L)	214 (182–258)	229 (185–298)	0.096
Creatinine (mg/dL)	0.73 (0.63–0.93)	0.93 (0.73–1.13)	<0.001 *
BUN (mg/dL)	14 (11–17)	18 (15–22)	<0.001 *
Glucose (mg/dL)	108 (96–125)	123 (103–146)	0.002 *
Lactate (mmol/L)	1.7 (1.2–2.3)	1.7 (1.1–2.2)	0.618
ALT (U/L)	41 (35–57)	34 (29–41)	0.020 *
Bilirubin (direct; mg/dL)	0.1 (0.10–0.13)	0.1 (0.10–0.20)	0.271
Troponin (ng/mL)	0.005 (0.001–0.010)	0.013 (0.005–0.034)	<0.001 *
**Electrocardiogram and heart rate variability indices—upon ED arrival**			
PR interval (ms)	156 (142–179)	158 (144–172)	0.308
QRS interval (ms)	82 (76–92)	86 (78–95)	0.170
QTC interval (ms)	411 (400–432)	413 (395–428)	0.552
SDNN (ms)	17.5 (11.1–29.7)	20.1 (12.4–34.3)	0.211
RMSSD (ms)	18.4 (12.0–30.8)	21.5 (13.3–33.1)	0.279

* *p*-value < 0.05. ALT—Alanine aminotransferase; BMI—Body Mass Index; BPM—Beats Per Minute; BUN—Blood Urea Nitrogen; RMSSD—Root Mean Square of Successive Differences; SDNN—Standard Deviation of NN Intervals; WBC—White Blood Cells.

**Table 2:** Multivariable logistic regression analysis for prediction of acute abdomen.

	AOR (95% CI)	*p*-Value
Age (years)	0.943 (0.904–0.983)	0.006 *
Pulse (BPM)	0.992 (0.953–1.031)	0.673
RMSSD (ms)	1.023 (1.004–1.043)	0.020 *

AOR—adjusted odds ratio; BPM—Beats Per Minute; RMSSD—root mean square of successive differences; * *p* < 0.05.

**Table 3:** Multivariable logistic regression analysis for prediction of acute myocardial infarction (MI).

	AOR (95% CI)	*p*-Value
Pulse (BPM)	1.052 (1.007–1.099)	0.023 *
Glucose (md/dL)	0.883 (0.809–0.965)	0.006 *
RMSSD (ms)	0.763 (0.605–0.962)	0.022 *

AOR—adjusted odds ratio; BPM—Beats Per Minute; RMSSD—root mean square of successive differences; * *p* < 0.05.

## Data Availability

The data presented in this study are available from the corresponding author upon reasonable request. The data are not publicly available due to privacy and ethical restrictions.
